# Supplementary material for: Learning curve and surgical outcome of robotic assisted colorectal surgery with ERAS program
Source: Sci Rep. 2022 Nov 29;12:20566. doi: 10.1038/s41598-022-24665-w (PMC9709162; doi:10.1038/s41598-022-24665-w)
Supplement: Supplementary file 2 — Supplementary Information 2. [file 41598_2022_24665_MOESM2_ESM.zip › RAL Raw data-Ñ[▒K/104/HS_ñw╝╨░O▒Kñσ.pdf]

正式摘要(電子簽章：0000002234341 66)

W95 / W95 ☐ 英文標題(注意:無英文姓名-請補病人基本檔!) ☐【讀取】憑證

# 臺中榮民總醫院

## 出院病歷摘要

轉入號： 轉出號：

[redacted]

| | |
|---|---|
| (8)流水編號 | |
| (9)入院日期 | 110/08/11 (2021) 直腸外科 W95 - 26 |
| (10)轉科日期 | |
| (11)出院日期 | 110/08/20 (2021) 住院天數計 9 日 |

## 診斷

- 入院 :1. Rectal cancer, adenocarcinoma with metastatic lymphadenopathies, cT3N1M1, RAS mut,
  - s/p IGRT-VB1 25 Gy/5 fr. (on 2021/02/22~02/26), s/p FOLFOXIRI + Avastin for 6 cycles (on 2021/03/05, 2021/03/25, 04/08, 04/22, 05/07, 05/21)
- 出院 :1. Rectal cancer, adenocarcinoma with metastatic lymphadenopathies, cT3N1M1, RAS mut,
  - s/p IGRT-VB1 25 Gy/5 fr. (on 2021/02/22~02/26), s/p FOLFOXIRI + Avastin for 6 cycles (on 2021/03/05, 2021/03/25, 04/08, 04/22, 05/07, 05/21)
  - r/o bilateral inguinal metastatic LNs, s/p bilateral inguinal lymph node dissection on 2021/07/16, s/p debridement on 2021/08/12
  - s/p Robotic-assisted low anterior resection on 2021/08/12

## 主訴

- admitted for scheduled surgery

## 病史

- This 40-year-old man has history of
  1. Rectal cancer, adenocarcinoma with metastatic lymphadenopathies, cT3N1M1, RAS mut,
  s/p IGRT-VB1 25 Gy/5 fr. (on 2021/02/22~02/26),
  s/p FOLFOXIRI + Avastin for 6 cycles (on 2021/03/05, 2021/03/25, 04/08, 04/22, 05/07, 05/21), r/o bilateral inguinal metastatic LNs, s/p bilateral inguinal lymph node dissection on 2021/07/16
  2. Cerebral palsy
  At last admission, he underwent per-anal excision of tumor and bilateral inguinal lymph node dissection on 2021/07/16. Pathology report showed adenocarcinoma of rectum, moderately differentiated and metastatic adenocarcinoma of left inguinal lymph node (2/13). Therefore, he was admitted for Robot Assisted Low Anterior Resection.

## 體檢發現

- r/o groin LN metastasis
  - General appearance :
    - easy looking , conscious clear , without cardiopulmonary distress
    - Vital signs自動帶入date 體溫(BT) 脈搏(P) 呼吸(R) 血壓(BP) 身高(BH) 體重(BW) BMI
      20210811 36.9℃ 107次/分 16次/分 127/86mmHg 154.8cm 64.8kg 27.0
    - Vital signs: B.P. 127/86 mmHg , P.R. 107 /min , R.R. 16 /min , B.T. 64.8 ℃
  - HEENT :
    - Eyes: no anemic conjunctiva, no icteric sclera, normal EOM
    - ENT: no otorrhea, no rhinorrhea, no erythematous mucosa, no injected tonsil, no deformity
  - Neck: supple, freely movable, trachea midline,no deformity, no engorged jugular vein , no palpable thyroid or lymph node enlargement
  - Chest:
    - Inspection: Grossly normal, symmetric and fully expansion, no accessory muscle used, no operation scar, no spider angiomata, no gynecomastia
    - Palpation: no palpable mass or nodule, no subcutaneous emphysema, no tenderness
    - Ausculation: clear breathing sound
  - Heart:
    - Percussion: normal heart size
    - Ausculation: regular heart beat, normal S1, S2, no gallop, no murmur
  - Abdomen:
    - Inspection: in
      - flat contour, symetrically, no scar, no stria, no dilated veins, no rashes
    - Ausculation: normoactive bowel sound, no abdominal bruits
    - palpation: soft, no muscle guarding or rigidity, no local tenderness, no rebounding pain, no palpable mass, no palpable pulsation
    - Percussion: no shifting dulllness, no hepatomegaly, no splenomagaly
  -

| Peripheral pulsation : | CA | BA | RA | FA | PA | PTA | DPA |
|---|---|---|---|---|---|---|---|
| Right side : | ++ | ++ | ++ | ++ | ++ | ++ | ++/+++ |
| Left side : | ++ | ++ | ++ | ++ | ++ | ++ | ++/+++ |

  - Genito-anus: no tenderness or palpable mass lesion or rectal shelf, normal external genitalia, normal anal tone, brown stool

### CRS CONDITION

- CEA: 0.84 ng/ml
- Imaging studies(Sono , CT of abdomen and chest Films):
  - 檢驗項目 :CT, PELVIS 報告日期 :1100524
    主 訴 :
    Result:Y Finding: Post IV contrast CT scan of the whole abdomen with oral contrast showed: > Asymmetrical wall thickening of rectum. Compatible to the image of rectal ca. Mild improvement in size as compared with previous CT 2021/2/17. > An enlarged lymph node about 1.3 cm in size at left inguinal area. Smaller as compared with previous CT 2021/2/17. > Mild Fatty liver > R/O small gallbladder polyp. > Left large renal cyst upto 6 cm. > pancreas, right kidney, spleen are without specific abnormality. > Small lymph nodes less than 1cm in size over para-aortic region are noted. > mild DJD changes over T-L spine Impression: > Compatible to the image of rectal ca. Mild improvement in size as compared with previous CT 2021/2/17. > Lymph node at left inguinal area. Smaller as compared with previous CT 2021/2/17. For other details, please see the above descriptions.
-
  - Lung

## 手術日期及方法

- 手術日期及方法20210812 Robot Assisted Low Anterior Resection
  20210812 Debridement for wound(deep)5-10cm
- 手術結果及發現 :
  - 1. ulcerative tumor, about 1 cm in size at 2 cm FAV. 2. Total meso excision was complet 3. IMA high ligation+ 4. distal margin 2 cm, Surefrom 45 x3 for rectum 5. air tie test neg. donut intact. penrose in pelvis. 6. loop ileostomy in RLQ

## 住院治療經過

- He underwent Robotic-assisted low anterior resection and debridement for poor healing wound of right inguinal area on 2021/08/12. After surgery, he had regained conscious and tolerated well under PCA control. We kept ERAS-based postoperative care protocol. Foley was removed on POD-1, but was re-inserted at the same night due to urine retention. He complained about skin lesions with itchy on fingerwebs of right hand. Pompholyx with candidasis was suspected, then we applied topical Mycomb cream and mycosten cream. Mill-like discharge from penrose drain, suspect chyle leakage was noted. We kept oil-free diet and kept close observation. Foley tube was removed successully on POD-4. There was improvement on penrose drainage amount and more clear color. He had fair wound condition and surgical stitches was removed on POD-7. Under his relative stable condition, he was discharged with penrose drainage tube inserted and was arranged further OPD follow-up.

## 合併症

- Chyle leakage noted from penrose drain

## 一般檢查

- 累積報告：

| DATE | TIME | ALB | TP | BIL.T | BIL.D | ALKP | AST | ALT | LDH | AMY | LIPASE | NH3 | Ferritin | UA |
|---|---|---|---|---|---|---|---|---|---|---|---|---|---|---|
| 1100812 | 1703 | | | 0.82 | 0.20 | | 19 | 29 | | | | | | |
| 1100816 | 0901 | 3.1 | | | | | | | | | | | | |

| DATE | TIME | WBC | RBC | HGB | HCT | MCV | PLT | BLST | NEUT | BAND | SEG | LYM | MON | EOS | BAS |
|---|---|---|---|---|---|---|---|---|---|---|---|---|---|---|---|
| 1100812 | 1702 | 14720 | | 13.5 | 41.6 | | 260 | | 87.1 | | | 7.5 | 5.2 | 0.1 | 0.1 |
| 1100816 | 0910 | 8200 | | 14.1 | 42.7 | | 280 | | 83.0 | | | 7.6 | 6.1 | 2.9 | 0.4 |

| DATE | TIME | NA | K | CL | BUN | CREAT | eGFR | CA | Mg | P | CRP | ESR | PCT | D-Dimer | LACTATE |
|---|---|---|---|---|---|---|---|---|---|---|---|---|---|---|---|
| 1100812 | 1703 | 145 | 3.5 | | 7 | 0.70 | 132.76 | 8.5 | | | | | | | |
| 1100816 | 0901 | 140 | 3.9 | | 5 | 0.74 | 124.51 | 8.8 | | | | | | | |

## 特殊檢查

## 病理報告

- 活體組織切片病理報告　手術日期：**110/08/12**　病理號：**S1024892**

診斷醫師:文美卿 報告日期:110/08/20
Pathologic diagnosis:
1. IMA root, robotic low anterior resection --- Positive for metastatic adenocarcinoma (1/4)
2. Distal cut end, robotic low anterior resection --- Negative for tumor.
3. Rectum, robotic low anterior resection ---
Status post pre-surgical treatment. Adenocarcinoma, moderately differentiated, in submucosa. The bilateral cut ends are free of tumor.
4. Paraortic LN, robotic low anterior resection --- Negative for metastasis (0/2)
5. Peritoneum, robotic low anterior resection --- Lipogranuloma. Negative for malignancy.
Ancillary study for diagnosis:
1. IHC stains for CMV is done on section G, and shows negative result.
2. IHC stains for PMS2 and MSH6 are done on section G. All above markers are preserved nuclear expression on tumor cells.
Prognostic and predictive factor:
1. Tumor size: 2.0 x 1.6 x 1.0 cm.
2. Depth of invasion: Invasion of submucosa
3. Histologic grade: G2: Moderately differentiated.
4. Angiolymphatic invasion: Present.
5. Perineural invasion: Not identified.
6. Tumor deposits (discontinuous extramural extension): Not identified.
7. Macroscopic tumor perforation: Not identified.
8. Surgical Margins: a) Proximal Margin: Uninvolved by invasive carcinoma, distance of tumor from margin: 1 cm. b) Distal Margin: Uninvolved by invasive carcinoma, distance of tumor from margin: 7 cm. c) Radial or Mesenteric Margin: Uninvolved by invasive carcinoma, distance of tumor from margin: 2.8 cm.
9. Lymph node status: Involved (1/25).
10. Extranodal involvement: Not identified.
11. Tumor regression grading S/P CCRT: residual cancer with evident tumor regression, but more than single cells or rare small groups of cancer cells (partial response, score 2).
12. Type of polyp in which invasive carcinoma arose: Not identified.
13. Additional pathologic findings: Not identified.
14. Pathological TNM Stage: ypT1N1a(According to the eighth edition, American Joint Committee on Cancer Staging Guidelines for Tumors).
15. TNM descriptors: y (Post-treatment).
Gross description:
The specimen consists of 1) IMA root, a piece of tan yellow soft tissue, 4.0 x 2.2 x 1.1 cm. 2) distal cut end, a piece of tan mucosa-covered soft tissue, 2.0 x 2.0 x 1.1 cm. 3) a segment of colon, 10.5 cm in length and 5.0 cm in proximal cut end circumference, 3.0 cm in distal circumference, and mesocolic/perirectal soft tissue. A gray firm lesion, 2.0 x 1.6 x 1.0 cm, is noted, 1 cm to the proximal cut end. On cross section, the mesocolic soft tissue was not involved. The both cut ends are free of tumor grossly. 4) paraortic LN, a piece of tan soft tissue, 2.6 x 1.0 x 0.5 cm. 5) peritoneum, 2 pieces of tan yellow soft tissue, up to 1.1 x 0.6 x 0.3 cm. Representative part for section: A) IMA root B) distal cut end C) paraaortic LN D) peritoneum E,F) proximal cut end G-K) tumor L) distal cut end M) non tumor colon N-S) Lymph nodes.
Note:
1. The interpretation of MMR panel and recommended studies are as below: (1) No loss of nuclear expression of MMR proteins: low probability of microsatellite instability-high (MSI-H) . (2) Loss of nuclear expression of MLH1 and PMS2: testing for methylation of the MLH1 promoter and/or mutation of BRAF is indicated (the presence of a BRAF V600E mutation and/or MLH1 methylation suggests that the tumor is sporadic and germline evaluation is probably not indicated; absence of both MLH1 methylation and of BRAF V600E mutation suggests the possibility of Lynch syndrome, and sequencing and/or large deletion/duplication testing of germline MLH1 may be indicated). (3) Loss of nuclear expression of MSH2 and MSH6: high probability of Lynch syndrome (sequencing and/or large deletion/duplication testing of germline MSH2 may be indicated, and, if negative, sequencing and/or large deletion/duplication testing of germline MSH6 may be indicated). (4) Loss of nuclear expression of MSH6 only: high probability of Lynch syndrome (sequencing and/or large deletion/duplication testing of germline MSH6 may be indicated). (5) Loss of nuclear expression of PMS2 only: high probability of Lynch syndrome (sequencing and/or large deletion/duplication testing of germline PMS2 may be indicated). (6) Please note: There are exceptions to the above IHC interpretations. These results should not be considered in isolation, and clinical correlation with genetic counseling is recommended to assess the need for germline testing.
2. 12 out of 19 perirectal lymph nodes have completely necrotic metastatic tumor without viable tumor cells, thus not regarded as positive lymph node.
3. This case has been peer reviewed by two doctors.
#T-59600_2 #M-82633_2 2 1105 B0SP0F
#T-C4560_2 #M-62009_2 2 1105 000000

## 其它

## 出院時情況

- 3.改門診治療

## 出院指示

| 藥名 | 劑量 | 劑量單位 | 用法 | 頻次 | 數量 | 數量單位 | 現況 | 天數 | 單位 |
|---|---|---|---|---|---|---|---|---|---|
| (口)Takepron OD tab 30mg | 1 | TAB | PO | QDAC | 7 | TAB | TH | 7 | 天 |
| Mag. Oxide tab | 2 | TAB | PO | TID | 42 | TAB | TH | 7 | 天 |
| Neomycin oint 28gm | | TUB | TOP | QD | 0 | TUB | TH | 1 | 瓶 |
| Ultracet tab | 1 | TAB | PO | Q6H | 28 | TAB | TH | 7 | 天 |
| 20g MY comb Cream | | TUB | TOP | QD | 0 | TUB | TH | 1 | 瓶 |

## 出院計劃

- 門診日期
  - date on 2021/08/25
- 衛教 :一、飲食攝取：1.少量多餐。2.禁食刺激性食物，如太辣、太膩，大量飲酒及抽太多煙、濃茶和咖啡。3.多攝取高蛋白食物，以加速傷口修復。如：新鮮瘦肉、魚、蛋等。4.並減少吃產氣食物可避免脹氣。如：牛奶、豆類、地瓜、蛋、洋蔥。二、術後活動：1.三個月內不可從事下列活動：騎馬、騎腳踏車、劇烈的跳舞、久坐及開車，以免骨盆充血。2.手術後 6 ? 8 週內避免提超過5KG之重物，若提物品可使用腹托或束腹帶以免震動所引起之疼痛。3.依個人的體力採漸進增加活動量，可先採輕工作，如無不適，體力許可再增加活動量。三、傷口護理：1.保持傷口清潔、乾燥，若有不慎弄濕傷口，可用清潔的毛巾或紗布拭乾。2.每日觀察傷口，是否有紅、腫、熱、痛，或不正常的分泌物。四、居家服藥：1.帶回的藥物，注意藥袋上的服用劑量、時間及注意事項。2.服用軟便劑若解便正常或有腹瀉現象時應停藥或減量。 五、出院後，若有下列情況需立即返院檢查：1.高燒不退。2.腹部疼痛厲害或嚴重腹脹嘔吐。3.傷口流血不止，或是有不正常之分泌物4.有其它問題，請與大腸直腸外科相關人員聯絡 TEL: 04-23592525 轉5161-3 或 6051, 6052

主治醫師 2062F 蔣鋒帆 / 住院醫師 2336L 洪浚嚴

**正式摘要(電子簽章：000000223434166)**

W95 / W95

doctorRank:B
